# Obstacles to prompt and effective malaria treatment lead to low community-coverage in two rural districts of Tanzania

**DOI:** 10.1186/1471-2458-8-317

**Published:** 2008-09-16

**Authors:** Manuel W Hetzel, Brigit Obrist, Christian Lengeler, June J Msechu, Rose Nathan, Angel Dillip, Ahmed M Makemba, Christopher Mshana, Alexander Schulze, Hassan Mshinda

**Affiliations:** 1 Dept. of Public Health and Epidemiology, Swiss Tropical Institute, P.O. Box, CH-4002 Basel, Switzerland; 2 Ifakara Health Institute, P.O. Box 53, Ifakara, Tanzania; 3 Novartis Foundation for Sustainable Development, WRO-1002.11.56, CH-4002 Basel, Switzerland; 4 Papua New Guinea Institute of Medical Research, P.O. Box 60, Goroka, EHP 441, Papua New Guinea

## Abstract

**Background:**

Malaria is still a leading child killer in sub-Saharan Africa. Yet, access to prompt and effective malaria treatment, a mainstay of any malaria control strategy, is sub-optimal in many settings. Little is known about obstacles to treatment and community-effectiveness of case-management strategies. This research quantified treatment seeking behaviour and access to treatment in a highly endemic rural Tanzanian community. The aim was to provide a better understanding of obstacles to treatment access in order to develop practical and cost-effective interventions.

**Methods:**

We conducted community-based treatment-seeking surveys including 226 recent fever episodes in 2004 and 2005. The local Demographic Surveillance System provided additional household information. A census of drug retailers and health facilities provided data on availability and location of treatment sources.

**Results:**

After intensive health education, the biomedical concept of malaria has largely been adopted by the community. 87.5% (78.2–93.8) of the fever cases in children and 80.7% (68.1–90.0) in adults were treated with one of the recommended antimalarials (at the time SP, amodiaquine or quinine). However, only 22.5% (13.9–33.2) of the children and 10.5% (4.0–21.5) of the adults received prompt and appropriate antimalarial treatment. Health facility attendance increased the odds of receiving an antimalarial (OR = 7.7) but did not have an influence on correct dosage. The exemption system for under-fives in public health facilities was not functioning and drug expenditures for children were as high in health facilities as with private retailers.

**Conclusion:**

A clear preference for modern medicine was reflected in the frequent use of antimalarials. Yet, quality of case-management was far from satisfactory as was the functioning of the exemption mechanism for the main risk group. Private drug retailers played a central role by complementing existing formal health services in delivering antimalarial treatment. Health system factors like these need to be tackled urgently in order to translate the high efficacy of newly introduced artemisinin-based combination therapy (ACT) into equitable community-effectiveness and health-impact.

## Background

Malaria kills more than one million people annually, mostly children under five years of age in sub-Saharan Africa. As part of an integrated approach to malaria control, the World Health Organization promotes prompt access to effective treatment for all episodes of malaria [1]. However, it can be safely assumed that of the 2.4 billion people (2005) living in low-income countries [2], only few have access to high quality health-care, including appropriate malaria-treatment [3-5].

Increased attention to the issue of access to treatment is required in the light of rolling-out highly efficacious artemisinin-based combination therapy (ACT). Lack of access is a complex issue that can be better understood when seen in the context of poverty, vulnerability and livelihoods [6]. Which obstacles to treatment are relevant – and should be addressed by interventions – depends to a large extent on the local setting [3]. As there is no one-size-fits-all solution, several strategies have been proposed and tested to improve access to malaria treatment. These include scaling-up home-based management [7,8], stronger involvement of the private sector [9,10], improving case-management in health facilities [11] as well as integrated approaches [12]. In addition, it is now widely acknowledged that no malaria-control strategy can be successful and sustainable without an increased investment in the local health system through which the interventions are to be channelled [13-15].

In order to develop targeted and cost-effective interventions, it is essential to add to the knowledge of treatment rates an understanding of the major obstacles to treatment access in a particular setting. For Tanzania, the 2004–5 Demographic and Health Survey (DHS) reported 58.2% antimalarial use in children under 5 years of age with a recent fever [16]. Yet, how many of these children received appropriate treatment, in a timely fashion and in the correct dose – or why others did not receive an antimalarial drug – is not elaborated upon in DHS surveys.

The studies presented here assessed the impact of local knowledge of malaria and treatment seeking practices on access to malaria treatment in a rural Tanzanian community. The research was carried out in the frame of a comprehensive intervention programme which aims to improve access to malaria treatment in rural Tanzania (ACCESS Programme) [17].

## Methods

### Study setting

Treatment seeking for fever episodes was studied in the districts of Kilombero and Ulanga, south-eastern Tanzania, in 2004 and 2005. The study area comprised the 25 villages of the local Demographic Surveillance System (DSS) [18] with a population of 74,200 in 2004, as well as the town of Ifakara, the district capital of Kilombero (2001 population census: 45,726 [19]). The area is predominantly rural and malaria transmission is high and perennial [20,21]. In 2004, there were 14 health facilities (9 public, 5 private/mission) in the DSS area, as well as one private clinic and one district hospital in Ifakara. Malaria accounted for roughly half of all outpatient visits in these facilities. Government and private health facilities in Kilombero were running on a cost-sharing scheme. In Ulanga, no user fees were charged in government facilities and a Community Health Fund (CHF) offered a form of risk-protection for members of the fund. A detailed description of the study area can be found elsewhere [17]. At the time of the study, the recommended first-line treatment for uncomplicated malaria was SP, the second line treatment amodiaquine; quinine was recommended as third-line treatment and for cases of severe malaria [22].

### Treatment seeking surveys

To investigate local understanding of malaria and treatment seeking behaviour for recent fever cases, a cross-sectional cultural-epidemiological community survey was conducted in the DSS area and Ifakara. We took a village-stratified random sample with the number of households sampled proportional to the total number of households in the village. A total of 318 households were drawn from the registered 16,220 households in the 25 DSS villages. Only households with at least one child under the age of five years were eligible.

Sampled households were visited by a DSS interviewer between May and August 2004, within the schedule of the routine DSS data collection. In all households in which a fever episode in the previous 14 days was reported, the patient or caretaker (if the patient was younger than 12 years) was interviewed. Patients who had not yet recovered were not included, as their options for treatment-seeking were not yet exhausted. They were instead advised to seek care from a health facility.

For Ifakara town no up-to-date household list was available as a sampling frame. The local administrative structure was used to establish a list of households and to perform a two-stage random sampling of 223 households. Every household in Ifakara was assumed to belong to a ten-cell (a group which was originally composed of ten households) and be represented by a ten-cell leader (*balozi*). Through visits to local government officials, a comprehensive list of all 329 ten-cell leaders in Ifakara was established. A random sample of 35 ten-cell leaders was then visited in order to establish a complete household list for their ten-cells. Six households per ten-cell were then randomly sampled. A household may only have been missed if it was not recorded by any ten-cell leader. We tried to avoid double-listing of households claimed by several ten-cell leaders by cross-checking the names of the household heads. Sampled households were visited by two trained interviewers in May 2004. The same inclusion and exclusion criteria as in the DSS villages applied.

Spatial data on household locations as well as socio-economic status calculations were obtained from the DSS database, which provided such information for 70% of all interviewed cases. In order to consider distance to the nearest point of care as a predictor for treatment access, we also used geo-spatial data of 16 health facilities and 498 drug selling shops (DSS and Ifakara), collected during a survey in May – June 2004 [23].

Additional information was derived from a longitudinal study on treatment seeking behaviour during the main farming season. This *shamba *cohort (in Swahili, *shamba *= farm) included a random sample of approx. 100 farming households from 10 randomly selected DSS villages which were followed-up during the main cultivation period between December 2004 and August 2005. Every month, interviews were done with recent fever cases, applying the same inclusion and exclusion criteria as in the cross-sectional community survey. A detailed description of this study can be found elsewhere [24].

As the sampling methodologies differed (cross-sectional vs. longitudinal; general population vs. only farming households), the two studies may not be equally representative of the general population. For the analysis of people's understanding of malaria it was assumed that there would be no difference between the two samples and both datasets were pooled for analysis. However, the treatment seeking and risk factor analysis for the *shamba *cohort study was done separately and presented elsewhere [24].

In both studies, field-workers used an Explanatory Model Interview Catalogue (EMIC) for data collection [25]. This semi-structured interview guide was developed on the basis of preceding focus-group discussions and further qualitative research on people's understanding and experience of malaria [26-28]. EMIC data comprised quantitative information and narratives of reported signs and symptoms ("patterns of distress"), perceived causes of the illness, and resulting treatment-seeking behaviour. Apart from the reported signs and symptoms, the data analysis took into consideration the name given to the illness by the respondent (illness label).

Oral informed consent was obtained from all study participants prior to the interviews. Ethical clearance for both studies was granted by the National Institute for Medical Research of the United Republic of Tanzania (NIMR/HQ/R.8a/Vol. IX/236, 16th September 2003).

### Statistical analysis

Epi Info 6 was used for random sampling procedures. Data were double entered in Microsoft FoxPro and Microsoft Access (Microsoft Corp.), and checked for coding errors and consistency. Statistical analysis was done with Intercooled Stata 9 (College Station, Texas, USA). For spatial analyses, MapInfo Professional 7.0 (MapInfo Corp., Troy, New York, USA) and ArcView GIS 3.3 (ESRI, Redlands, CA, USA) were used.

For cultural-epidemiological data on patterns of distress (PD) and perceived causes (PC), answers were categorized and given values according to whether they were reported spontaneously (value of 2) or upon probing (value of 1). From these values, means were calculated as a measurement of the prominence of the respective features in the interviewee's accounts. Similar PD and PC were grouped for analysis and ranked according to their prominence in the interviewee's accounts. The Kruskal-Wallis test was used to test differences in the ranked outcomes between sub-groups of the sample. Chi^2 ^and Fisher's exact tests were used to test associations.

Univariate and multivariate logistic models were fitted to assess the effect of several predictors on prompt and effective treatment, as defined below. Univariate analysis was conducted for all possible predictors on which data had been collected within the frame of the programme. For the multivariate models, a stepwise backward estimation was performed with a P > 0.2 significance level for removal from the model. The likelihood ratio test was used for significance testing. Both analyses considered village vs. town as predictor, but not individual villages. The sampling strategy of households as described above was unlikely to have resulted in over-sampling of any of the villages. We therefore performed aggregate level analyses which should give unbiased estimates without any village weighting. In addition, the number of individuals from each village was very small and hence estimating or allowing for within-village correlation factors in the regression analysis would be meaningless.

## Results

### Study sample

In the cross-sectional community survey, 154 recent fever cases were identified (approximately 28% of all sampled households). In order to establish a clear distinction between cases in children and cases in adults, we only considered the cases aged under five years (80 children) and over 12 years (57 adults) for our analysis. The *shamba *survey added another 29 children under five years and 28 adults over 12 years to the sample. Basic characteristics of the sample are summarized in Table 1.

**Table 1:** Sample characteristics

**Characteristics**	**2004 community survey (N = 137)**		**2005 *shamba *survey (N = 57)**	
	**n**	**Percentage**	**n**	**Percentage**
***Age group***				
under 5 years	80	58.4	29	50.9
over 12 years	57	41.6	28	49.1

***Sex***				
Female	76	55.5	25	43.9
Male	61	44.5	32	56.1

***Residence***				
Ulanga DSS villages	53	38.7	31	54.4
Kilombero DSS villages	44	32.1	26	45.6
Ifakara	40	29.2	NA	NA

***Religion (of caretaker if patient < 12 years)***				
Muslim	51	37.2	21	36.8
Christian	84	61.3	36	63.2

***Years of formal education (of caretaker if patient < 12 years)***				
Mean (years)	5.4 (95% CI 4.83, 5.89)		6.5 (95% CI 6.01, 6.97)	
Median (years)	7 (51.1% of sample)		7 (77.2% of sample)	

***Household income regular and dependable***				
Yes	68	49.6	33	57.9
Possibly	18	13.1	3	5.3
Uncertain	13	9.5	7	12.3
No	38	27.7	14	24.6

### Local understanding of febrile illness

The analysis of patterns of distress and perceived causes considered all children under the age of five years and adults over 12 years (194 in total).

Based on the illness labels given by the interviewees, fever cases were classified into three illness categories that roughly correspond with biomedical malaria. Of the child cases, 68 (62%) were labelled *malaria*, 24 (22%) *homa *(literally: fever), and 8 (7%) *degedege *(convulsions, usually known only as an illness of young children). Of the adult cases, 54 (64%) were labelled *malaria*, 26 (31%) *homa*, and 2 (2%) *degedege*. This classification is popular in the community as has been described in detail in earlier qualitative studies from Tanzania [26,28,29]. *Homa *and *malaria *cases were more relevant for this analysis than the relatively rare *degedege*-labelled cases. We included the *degedege *cases reported in children, but excluded the two adult cases. A detailed list of PD and PC variables with corresponding prominence values is available as an online supplement to this paper [see Additional file 1].

#### Patterns of distress (PD)

In **children**, fever symptoms and loss of strength were most prominently mentioned. "Having no strength" was significantly more prominent in the *malaria *compared to the *homa *category (P = 0.023), as was vomiting (P = 0.033). Vomiting was generally more prominent than diarrhoea. Signs and symptoms related to convulsions (such as twitching, stiff body, "eyes turn white", kicking of arm or leg, froth in the mouth, mouth twisted sideways, as well as delirium, falling down, and being easily startled or frightened) were most prominent in the *degedege *category. Twitching was significantly more prominent in the *degedege *than the *malaria *(P = 0.029) and the *homa *categories (P = 0.022). 68 (62.4%) cases showed at least one of the aforementioned signs of convulsions. Of these, 44 (61.1%) were labelled *malaria*, 15 (20.8%) *homa*, and 7 (9.7%) *degedege*, showing clearly that many caretakers make a link between convulsions and malaria. Respiratory symptoms were not very prominent [see Additional file 2 – Figure A1].

In **adults**, there was no clear difference in reported PD between *homa *and *malaria *cases. Symptoms related to body strength or pain (particularly headache) were at least as prominent as fever. Nausea and vomiting were more prominent than diarrhoea. Difficult breathing or cough were less prominent in adults than in children. Convulsion-symptoms, such as twitching, were not probed – but also not mentioned spontaneously [see Additional file 2 – Figure A1].

#### Perceived causes (PC)

Fever cases in **children **were most commonly attributed to mosquito bites. A "bird or insect called *degedege*", previously reported to be seen as a cause of *degedege *[29] was not significantly associated with *degedege *nor with signs of convulsions. The physical constitution of a child was rather seen as a cause of *homa *than *malaria *(P = 0.023). *Homa *rather than *malaria *was often seen as a "stage of child growth" (P = 0.033). Cold weather as a cause was most prominent in the *homa*, and least prominent in the *malaria category *(P = 0.044). Heat as well as cold weather, was prominently mentioned as a cause of *degedege*. Supernatural causes (God, spirits, sorcery, etc.) were rarely mentioned. However, of all these, God was mentioned prominently in the *malaria *category, just after mosquito bites [see Additional file 2 – Figure A2].

Also in **adults**, mosquito bites were mentioned most prominently. Physical constitution was rather seen as a cause of *homa *than *malaria*. Sanitation or a dirty environment were more prominent in the *malaria *category (P = 0.040), while climate-related causes were more prominent in the *homa *category (P = 0.057). God was the most prominently mentioned supernatural cause [see Additional file 2 – Figure A2]. A more detailed description of local illness concepts can be accessed elsewhere [30].

### Help seeking for fever episodes

The analysis of help seeking for a recent febrile illness episode considered only the 2004 cross-sectional community survey data.

#### Immediate help-seeking action

**Figure 1:**
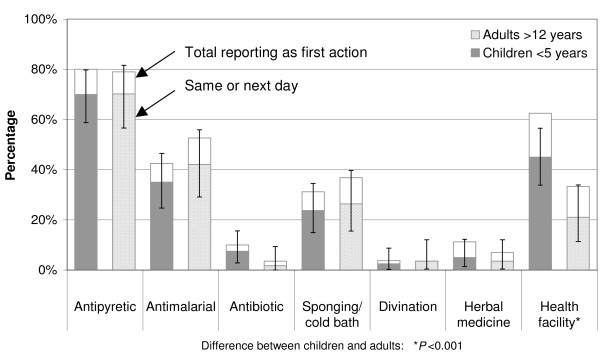
**Immediate help seeking actions taken on the day of illness onset or the day after.** Error bars represent 95% confidence intervals.

Once a fever episode was recognized, most children and adults took an antipyretic drug, about 70% of them on the day of illness onset or the day after. Antimalarial drugs were less popular as first help seeking action: only 35.0% (24.7–46.5) of all children and 42.1% (29.1–56.0) of all adults took an antimalarial on the same or the next day. 62.5% of all caretakers brought their sick child to a health facility as first action, 45.0% (33.9–56.5) of them within two days. Significantly fewer adults attended a health facility as first action (33.3%) and only 21.1% (11.4–33.9) went there within two days. Cooling one's body through sponging or a cold bath was done by over 30% of all children and adults. Traditional medicine or divination, however, was not common (Figure 1).

Interestingly, even in child-cases labelled *malaria*, antipyretic administration (78.8%, 65.3–88.9) was more frequent as first action than administration of antimalarials (48.1%, 34.0–62.4). At the same time, the frequency of antimalarial administration was not significantly different between the illness labels or between cases with (44.7%, 29.9–59.4) or without convulsions (39.4%, 21.8–57.0). However, herbal medicine was given significantly more often to children with *degedege *than to children with *homa *or *malaria *(P = 0.020). For children, there was a significant difference between the areas of residence (P = 0.007) with antimalarials being administered most frequently in Ulanga DSS (60%) and less frequently in Kilombero DSS (39%) and Ifakara (18%).

In adult cases, administering antipyretics was the most frequent help seeking action for both *homa *and *malaria*. Antimalarials were taken more frequently for *malaria *than for *homa *(P = 0.028). A cold bath or shower was significantly more frequent in the *malaria *(51.4%, 34.0–68.6) than the *homa *(10.5%, 1.3–33.1) category (P = 0.003). Significantly more adults went to a health facility in Ifakara than in the DSS villages of Kilombero (P = 0.041). However, no significant difference between the illness labels was seen in health facility attendance rates.

#### Sources and appropriateness of malaria treatment

In order to estimate population coverage with prompt and appropriate malaria treatment ("effective coverage"), key treatment indicators were assessed, including all reported treatment steps (Table 2). An almost 100% usage of biomedical treatment was noted in adults and children. Antimalarial administration to children was common (88.8%) with the majority receiving quinine (53.8%). 34.9% of the children who received quinine, were given an injection or infusion. Less than half of the children and adults received the first-line drug SP. Most of the SP treatments given to children were wrongly dosed. 28.8% (95% CI 19.2–40.0) of the children and 12.3% (5.1–23.7) of the adults were treated with more than one product, most commonly with two (P = 0.022).

**Table 2:** Key indicators for help seeking and access to malaria treatment in individuals with fever in the preceding two weeks

**Indicator**	**Children** **(N = 80)**			**Adults** **(N = 57)**		P*
	**n**	**% (95% CI)**	**TDHS**^**2**^	**n**	**% (95% CI)**	
Episodes treated	80	100 (95.5–100)		57	100 (93.7–100)	

***Medications and dosaging:***^1^						
Modern medicine	80	100 (95.5–100)		56	98.3 (90.6–100)	0.416^∞^
Antimalarial drug (AM)	71	88.8 (79.7–94.7)	58.2 (88.6)	47	82.5 (70.1–91.3)	0.293^§^
- SP	38	47.5 (36.2–59.0)	23.7 (33.8)	25	43.9 (30.7–57.6)	0.673^§^
- SP correctly dosed	13	16.25 (8.9–26.2)		18	31.6 (19.9–45.2)	**0.035**^§^
- amodiaquine	10	12.5 (6.2–21.8)	22.1 (29.3)	5	8.8 (2.9–19.3)	0.491^§^
- amodiaquine correctly dosed	5	6.3 (2.1–14.0)		3	5.3 (1.1–14.6)	1.000^∞^
- quinine	43	53.8 (42.2–65.0)	11.9 (23.5)	23	40.4 (27.6–54.2)	0.122^§^
- other AM	2	2.5 (0.3–8.7)		1	1.8 (0.0–9.4)	1.000^∞^
Antipyretic only	9	11.3 (5.3–20.3)		9	15.8 (7.5–27.9)	0.438^§^
Antibiotic	8	10.0 (4.4–18.8)		2	3.5 (0.4–12.1)	0.194^∞^

***Treatment sources:***^1^						
Health facility visit	61	76.3 (65.4–85.1)		32	56.1 (42.4–69.3)	**0.013**^§^
AM from health facility	43	53.8 (42.2–65.0)		17	29.8 (18.4–43.4)	**0.005**^§^
AM not from health facility	28	35.0 (24.7–46.5)		30	52.6 (39.0–66.0)	**0.040**^§^
AM from drug store	19	23.8 (15.0–34.6)		26	45.6 (32.4–59.3)	**0.007**^§^
AM from general shop	8	10.0 (4.4–18.8)		4	7.0 (2.0–17.0)	0.761^∞^
AM from home stock (or relative/neighbour)	10	12.5 (6.2–21.8)		6	10.5 (4.0–21.5)	0.723^§^
Exclusive home-management with AM^3^	14	17.5 (9.9–27.6)		18	31.6 (19.9–45.2)	0.055^§^

^1^One episode may be treated with several drugs from various sources; ^2^Tanzania Demographic and Health Survey 2004–05 (data for Morogoro Region in brackets); ^3^Episodes never brought to a health facility; * comparison of children and adults; ^∞^Fisher's exact test; ^§^Chi-square test

Few episodes were treated with an antipyretic only, or with an antibiotic. Overall, children (76.3%) were more often brought to a health facility than adults (56.1%). Children (53.8%) were also more likely to receive their antimalarial from a health facility than adults (29.8%). Adults rather opted for non-exclusive (P = 0.040) or exclusive home-management with antimalarials than children. Generally, drug stores were the most important source for home-treatment, particularly for adults. However, of the 57 children who were brought to a health facility and received an antimalarial, 24.6% obtained the drugs from a source other than the facility.

Timeliness of treatment with an antimalarial was significantly better in children than in adults. 76.3% (65.4–85.1) of the children, but only 56.1% (42.4–69.3) of the adults received an antimalarial on the day the fever started or the day after (P = 0.013). When adjusted for age group (adults vs. children), those who obtained their antimalarial from a health facility were more likely to receive it on the day the illness started (P = 0.004).

#### Effective community coverage

**Figure 2:**
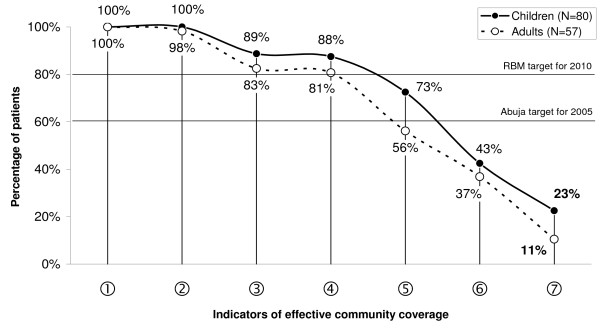
**Estimated effective coverage of fever treatment modelled based on patients' or caretakers' accounts. Percentages are proportions of the study sample with a reported recent fever**. ① Episode treated. ② Drug administered. ③ Antimalarial administered. ④ Recommended antimalarial. ⑤ Recommended antimalarial on same or next day. ⑥ Recommended antimalarial on same/next day, in correct dose. ⑦ Recommended antimalarial on same/next day, correct dosage, appropriate considering reported symptoms.

Effective community coverage with prompt and appropriate malaria treatment was estimated based on the reported treatment-seeking behaviour for recent fever episodes, taking into account the national treatment guidelines [22]. The main indicators are shown in Figure 2, illustrating how dramatically effective coverage is reduced because of weaknesses in the treatment chain. 87.5% (78.2–93.8) of the children and 80.7% (68.1–90.0) of the adults received one of the antimalarials recommended by the national guidelines (SP, amodiaquine or quinine) [22](④). 72.5% (61.4–81.9) of the children and 56.1% (42.4–69.3) of the adults received these drugs on the day of onset of the symptoms or the day after (⑤). 42.5% (31.5–54.1) of the children and 36.8% (24.4–50.7) of the adults received the antimalarial not only in time, but also in the recommended dose (⑥). Dosage was assessed based on the patient's or caretaker's accounts. Due to a lack of detailed information, it was assumed that all injections were correctly dosed. Most wrong dosages were under-dosages. If also the reported symptoms are taken into account, only 22.5% (13.9–33.2) of the children and 10.5% (4.0–21.5) of the adults received timely treatment with an appropriate and correctly dosed antimalarial. SP or amodiaquine was considered appropriate for reported symptoms of uncomplicated malaria, quinine if severe symptoms (incl. difficult breathing, yellow eyes, convulsions, delirium [22,31]) had been reported.

#### Expenditures for antimalarials

Expenditures for antimalarials of patients exclusively attending either a health facility or a drug store are presented in Table 3. Children and adults paid similar prices in health facilities and in drug stores. Although children should be treated free of charge in public health facilities, they paid on average 540 Tanzanian Shillings (TShs) for antimalarials obtained in health facilities. It was not possible to distinguish between private and public health facilities in this analysis. However, there was no difference between households from villages with either a public or a private health facility, suggesting that exemption mechanisms were not properly implemented. Quinine (median price TShs 570, interquartile range [IQR] 0–1100) was sold frequently and was on average more expensive than SP (TShs 300, IQR 0–500), contributing substantially to total drug expenditures.

**Table 3:** Median reported expenditure per one dose of antimalarial treatment in health facilities and drug stores (in TShs). US $1 = TShs. 1,117 (July 2004)*

	**Children**			**Adults**		
	
	**n**	**Median expenditure** **(IQR)**	**Range**	**n**	**Median expenditure** **(IQR)**	**Range**
Health facility	35	540 (0–1100)	0 to 3000	15	500 (200–700)	0 to 2700
Drug store	16	600 (400–900)	0 to 3600	21	540 (400–800)	60 to 2000

* OANDA currency converter

IQR = Interquartile range

#### Factors related to prompt and effective treatment

**Figure 3:**
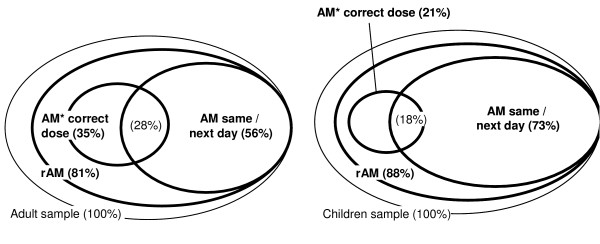
**Graphical illustration of treatment indicators assessed in the multivariate models**. Circles are roughly proportional to the percentage of patients. rAM = recommended antimalarial (SP, amodiaquine, quinine). * includes only SP and amodiaquine.

Three multivariate logistic models were fitted to assess predictors for the administration of a recommended antimalarial, as well as for timely and correctly dosed treatment (Figure 3). Models were fitted once for the DSS only and once for both the DSS and Ifakara households. Spatial information and SES were only available for the DSS households.

Fever cases in DSS households were less likely to receive a recommended antimalarial if their illness was labelled *homa *(fever) (OR = 0.08), they received traditional herbal medicine as first action (OR = 0.08) or they lived in a household with a higher total number of people (OR = 0.79). On the other hand, attending a health facility during the course of the illness (OR = 7.69) increased the odds of receiving a recommended antimalarial (Table 4). When this model was fitted with DSS and Ifakara households, only the effects of the *homa*-label and the number of people in the household were retained. In the univariate analysis, recognising the illness while working in the fields (*shamba*) and increased distance to the nearest antimalarial provider were also significantly correlated with less antimalarial administration, while reported diarrhoea or vomiting significantly increased the odds. Episodes in DSS households were less likely to receive a recommended antimalarial than those in Ifakara.

**Table 4:** Univariate and multivariate analyses of predictors for administration of a recommended antimalarial (SP, amodiaquine or quinine)

	Univariate model*			Multivariate model**		
	
**Exposure variable**	**Crude OR**	**95% CI**	**P**	**Adjusted OR**	**95% CI**	**P**
**Age group**						
Adult (> 12 years)	1					
Child (< 5 years)	1.67	0.66–4.26	0.280			
**Total number of people in household**	0.85	0.73–0.99	0.041	**0.79**	**0.64–0.97**	**0.021**
**Place of illness recognition**						
Home	1			1		
Shamba	0.19	0.07–0.51	0.001	0.29	0.08–1.14	0.076
**Diarrhoea or vomiting reported**						
No	1			1		
Yes	5.05	1.73–14.74	0.003	3.24	0.85–12.34	0.084
**Signs of severe malaria**						
No						
Yes	2.48	0.78–7.88	0.125	3.60	0.73–17.86	0.117
**Illness label (self-defined)**						
Malaria/degedege	1			1		
Homa	0.10	0.04–0.29	0.000	**0.08**	**0.02–0.32**	**< 0.001**
**First action: Antipyretic**						
No	1					
Yes	0.61	0.17–2.23	0.451			
**First action: Traditional medicine**						
No	1			1		
Yes	0.24	0.07–0.81	0.022	**0.08**	**0.01–0.48**	**0.006**
**Health facility attendance**						
No	1			1		
Yes	4.46	1.69–11.78	0.003	**7.69**	**1.90–31.11**	**0.004**
**Antimalarial provider in village**^**1**^						
No	1					
Yes	2.10	0.67–6.59	0.201			
Distance to nearest antimalarial provider (km)***^/1^	0.01	0.00–0.43	0.017			
**Study area**						
Ifakara	1					
DSS	0.22	0.05–0.98	0.046			

* 137 observations; ** 136 observations; *** 76 observations (DSS only); ^1 ^Incl. health facilities, drug stores and general shops stocking antimalarials in mid-2004

The second model assessed predictors for timely treatment (i.e. on the day of illness onset or the day after) among all those receiving a recommended antimalarial. None of the plausible predictors was significantly correlated with the outcome with the exception of a higher number of people in the household, which decreased the odds of timely malarial treatment (OR = 0.82, 0.69–0.98). The age of the patient, distance to the nearest provider, or prior treatment with other medicines were not found to be significant predictors of the outcome.

The third model assessed predictors for correct dosage among those receiving SP or amodiaquine. This analysis did not consider quinine, as it was not possible to establish the accuracy of the dosage of quinine injections. In this model, children's odds of receiving the correct dose was significantly decreased (OR = 0.26, 0.09–0.75) compared to adults. Cases from DSS households were more likely to receive a correctly dosed drug than those from Ifakara (OR = 4.44, 1.27–15.51).

## Discussion

This paper assessed population coverage with prompt and effective malaria treatment from the perspective of those affected. The analysis revealed some important issues for the development of future malaria control strategies. However, in order to establish a comprehensive understanding of access to health, it may be useful to combine several approaches, as elaborated by Obrist *et al*. [6].

Local concepts of malaria will influence successful implementation of effective case-management [32]. This research demonstrated clear changes in the understanding of *degedege *as compared to historical data. *Degedege *was traditionally linked to spirits in the form of a bird or a moth and mainly herbal treatment would be administered by traditional healers [29,33]. Studies done between 1995 and 1997 in the same area found that concepts of *degedege *and *malaria *were fuzzy; while *degedege *was sometimes seen as a cause of severe malaria, only mild malaria would be related to mosquito bites [27,34]. Today, fevers with convulsions were in most cases labelled *malaria *rather than *degedege*. Mosquito bites were usually seen as the cause for convulsions and *degedege*. These findings are currently being validated through an in-depth study on *degedege *cases. In contrast to *degedege*, which was considered a severe and dangerous illness [34], *homa *("fever") was often regarded as a normal stage in child growth or was attributed to weather conditions. Similar findings have been reported from other regions of Tanzania [28]. While for *homa *and *malaria *different symptoms were reported in children, this was not the case in adults. It also appeared that *malaria *was usually associated with mosquitoes whereas *homa *was often attributed to other causes. In earlier studies, *homa *has been described as a label for general malaise and aches, sometimes even in the absence of fever [35,36]. To some extent this may explain why fever as a symptom was not most prominent in all *homa *cases. Nevertheless, local illness labels may still influence treatment-seeking behaviour. As a result, those cases labelled *homa *were more than 12 times (OR = 0.08) less likely to receive an antimalarial than cases labelled *malaria *or *degedege*.

An increasing overlap of the popular and biomedical concepts of malaria can be attributed partly to regular and intensive health education campaigns in the area, from the national "Mtu ni Afya" (Man is Health) campaign in the late 1970s [26] to the intensive social marketing of insecticide-treated nets in the 1990s [27,37]. Nevertheless, factual knowledge does not necessarily translate directly into improved behaviour [34], particularly since appropriate care-seeking depends on several factors other than illness understanding.

In contrast to what has been reported from earlier studies and other areas [38-40], no fever episode remained untreated. Antimalarial administration was common, even for cases with convulsions or labelled *degedege *and traditional herbal medicine use was rare. This is a major improvement in treatment seeking when compared to a household survey carried out in two DSS villages in 1995–97, which reported 35% of *degedege *episodes were treated with herbal medicine and only 2% received an antimalarial [26]. It fits well with the findings of de Savigny *et al*. [41] who reported 78.7% of sufferers first use biomedical care for cases of fatal malaria in Tanzania. Considering that first treatment with herbal medicine was correlated with less antimalarial use (OR 0.08), discouraging herbal treatment may help to increase treatment rates with antimalarials.

The frequent usage of antipyretics as first treatment may partly reflect a decreased availability of antimalarials in shops [23] and the Tanzanian malaria control policy, which does not actively promote home-based management with antimalarials. Most of the children were brought to a health facility, which increased the chance of receiving a recommended antimalarial. Health facility attendance is generally desirable because other severe febrile illnesses, such as pneumonia or meningitis, can not easily be managed at home. Despite high health facility usage rates, however, the proportion of patients receiving an appropriate antimalarial timely and correctly dosed (23% of all children and 11% of all adults) was low and too far from the 80% target set by the RBM Partnership for 2010 [42]. These figures put into perspective the 51.1% use of a recommended antimalarial on the same or next day for Tanzania and 82.7% for Morogoro Region reported by the DHS [16]. At the same time, the DHS figures also validate the results of some of our indicators, except for the rates of quinine use, which were extraordinarily high in our study.

Patients, particularly children, who were treated at a health facility, were not more likely to receive an appropriately dosed antimalarial. Reported wrong dosages may be attributed to poor patient adherence or reporting errors. Yet, without any doubt, quality of care is a critical step in assuring appropriate treatment. Merely limiting antimalarial sales to health facilities and drug stores [23] has apparently not resulted in an acceptable quality of case-management. The observed effect of the number of people in a household on (timely) antimalarial treatment may be a chance finding or related to intra-household relations [43]. Yet it may also reflect that in larger families, child care is often delegated from parents to older siblings who may not yet know how to handle an illness episode. In families with more children (or elderly household members), caretakers may be more busy and have less time devote to each one.

Children under 5 years of age should be treated free of charge in (government) health facilities. Yet 25% of the child episodes seen at a health facility were eventually treated with a shop-bought antimalarial. One might expected that commercial sector treatment of childhood fevers would pose an additional and unnecessary burden on poor households since in the private sector no exemption policy applies. Njau *et al*. reported significantly higher spending in drug stores (but not general shops) compared to government health facilities [44]. However, data from this study suggests that due to a dysfunctional exemption system drug expenditures did not significantly differ between health facilities and drug stores. In this situation, it may even be cheaper to treat a child with shop-bought drugs. A shorter distance to shops than health facilities may result in lower secondary costs for time and transport. This may consequently contribute to higher facility usage rates and possibly to more use of antimalarials by richer households as reported elsewhere [45]. This link, however, could not be proven in our study.

Stock-outs of SP, amodiaquine, and quinine, which were repeatedly observed in the study area [46], may have forced certain patients to purchase medicines from shops. The commercial sector therefore plays a pivotal role in providing life-saving drugs in the case of delivery-failure of formal health facilities.

The key issues resulting from this research need to be interpreted in consideration of the research methodology and the study setting. Case-selection was based on reported fever rather than lab-confirmed malaria, acknowledging that due to a lack of diagnostics in most primary health facilities episodes of fever suggestive of malaria are treated as such. The Integrated Management of Childhood Illness (IMCI) algorithms (which are implemented in the study area), as well as the national malaria treatment guidelines advocate an assessment and treatment based on clinical signs and symptoms if no microscopy is available [22]. Very often however, a diagnosis is based on the patient's or caretaker's reported symptoms rather than a proper clinical assessment [47,48]. The study generally relied on patients' or caretakers' accounts which may result in misreporting of symptoms and treatment seeking behaviour. However, in real life, it is also the patient's perceived ill-health that triggers help-seeking action, rather than a clinical or laboratory diagnosis [4].

When making inferences to other areas one should consider the limited sample size of the studies and the long history of malaria-control and research activities in the study area. The latter would suggest that treatment and coverage rates in the area are likely to be above the Tanzanian average.

It is also worth considering that all estimates presented here did explicitly exclude the aspect of drug efficacy. Yet, actual efficacy of SP in children in nearby Mlimba village was reported to be only 65.7% (adequate clinical and parasitological response at day 28) [49]. Furthermore, 24 % of SP tablets and 40 % of quinine sulphate tablets collected in the study area did not meet USP specifications for the amount of active ingredient and were mostly under-dosed [17,50]. Adding these factors into an effectiveness-model would result in even lower levels of community effectiveness than reported in the coverage figures above.

In the ongoing process of rolling out highly efficacious artemisinin-based combination therapy (ACT), special attention will have to be paid to the quality of prescription in formal health facilities, the ability and willingness of patients to comply with the treatment regimen, as well as to the channels through which these drugs are brought to the patients. It needs to be closely monitored whether restricting ACT to the formal health sector will result in increased community effectiveness (timely administration of correctly dosed efficacious ACT to all those in need) or in a decrease in treatment rates without an improvement in the administered drug regimens.

## Conclusion

In the study area, the local understanding of the biomedical concept of malaria has markedly improved after continuous health education. Further education may have to focus on how, when and where to treat a febrile illness instead of reiterating illness concepts. Availability of antimalarials has been largely limited to certified providers and health facility usage was very popular. Nevertheless, the quality of case-management was far from satisfactory. Decreased drug efficacy and sub-standard drug quality may have further decreased effectiveness at community level. Exemption mechanisms aim to facilitate treatment access for poor and vulnerable groups but in our setting they did not seem to be properly implemented. Private drug retailers played a central role in the provision of timely malaria treatment, complementing the existing formal health services. All these issues can be attributed to a health system which is still too weak to deliver a growing number of increasingly complex health interventions. Such constraints need to be tackled urgently with an increased investment in the local health system in order to translate the high efficacy of newly introduced ACT into equitable community-effectiveness and health-impact.

## Competing interests

The authors declare that they have no competing interests.

## Authors' contributions

MWH was involved in the design and implementation of the studies, analysed and interpreted the data and wrote the manuscript. BO and CL conceived the studies and contributed to the writing, interpretation and discussion of the manuscript. JJM developed the EMIC together with BO and MWH, and was responsible for its implementation. RN was in charge of all DSS-related activities. AD contributed to the analysis of the data. AM and CM provided support in the field and contributed to the discussion of the findings. AS and HM contributed to the design of the study and to the discussion of the manuscript.

## Pre-publication history

The pre-publication history for this paper can be accessed here:



## Supplementary Material

Additional file 1 **Mean prominence values for patterns of distress (PD) and perceived causes (PC) in children and adults.** Click here for file

Additional file 2 **Graphical illustration of patterns of distress (PD) and perceived causes (PC) by illness category**. Red arrows point out significant differences between the categories. *Figure A1: *Patterns of distress. Bars represent grouped reported PD. PD with the highest mean prominence values are listed as most prominent PD. *Figure A2: *Perceived causes. Bars represent grouped PC. PC with the highest mean prominence values are listed as most prominent PC. Click here for file
