# Supplementary material for: Obstacles to prompt and effective malaria treatment lead to low community-coverage in two rural districts of Tanzania
Source: BMC Public Health. 2008 Sep 16;8:317. doi: 10.1186/1471-2458-8-317 (PMC2564938; doi:10.1186/1471-2458-8-317)
Supplement: Additional file 2 — Graphical illustration of patterns of distress (PD) and perceived causes (PC) by illness category. Red arrows point out significant differences between the categories. Figure A1: Patterns of distress. Bars represent grouped reported PD. PD with the highest mean prominence values are listed as most prominent PD. Figure A2: Perceived causes. Bars represent grouped PC. PC with the highest mean prominence values are listed as most prominent PC. [file 1471-2458-8-317-S2.pdf]

Hetzel *et al.* Obstacles to prompt and effective malaria treatment lead to low community-coverage in two rural districts of Tanzania.

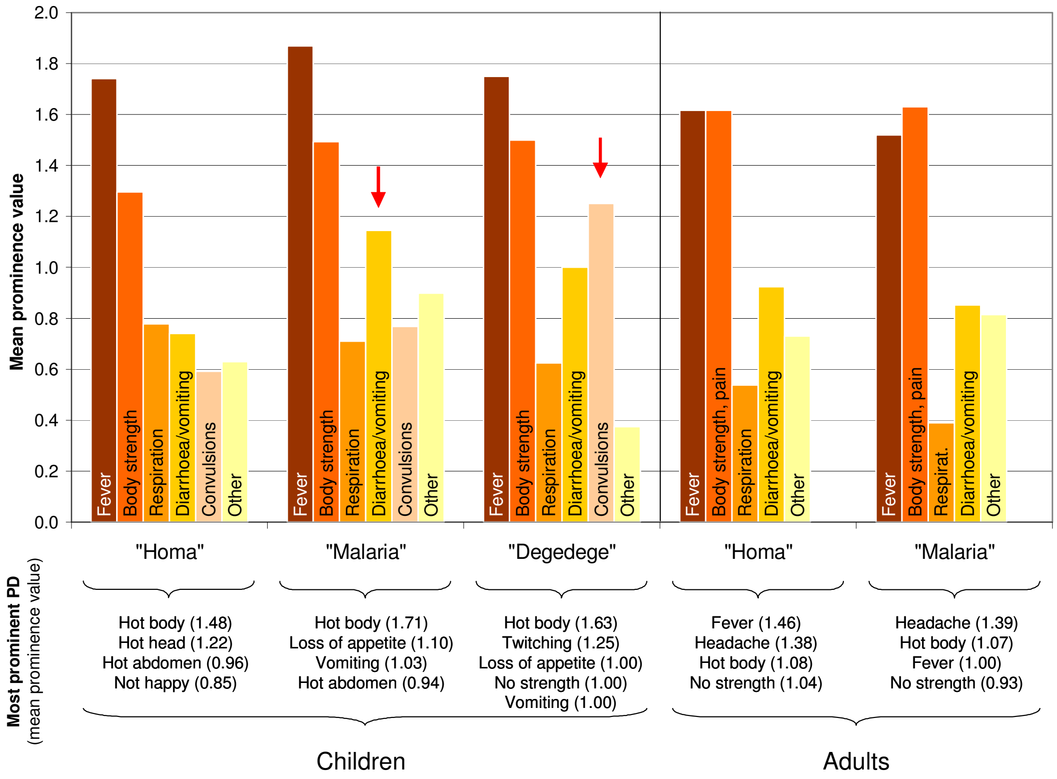

Figure A1: Pattern of distress (PD) by illness category. Grouped (bars) and most prominent signs and symptoms.

Hetzel *et al.* Obstacles to prompt and effective malaria treatment lead to low community-coverage in two rural districts of Tanzania.

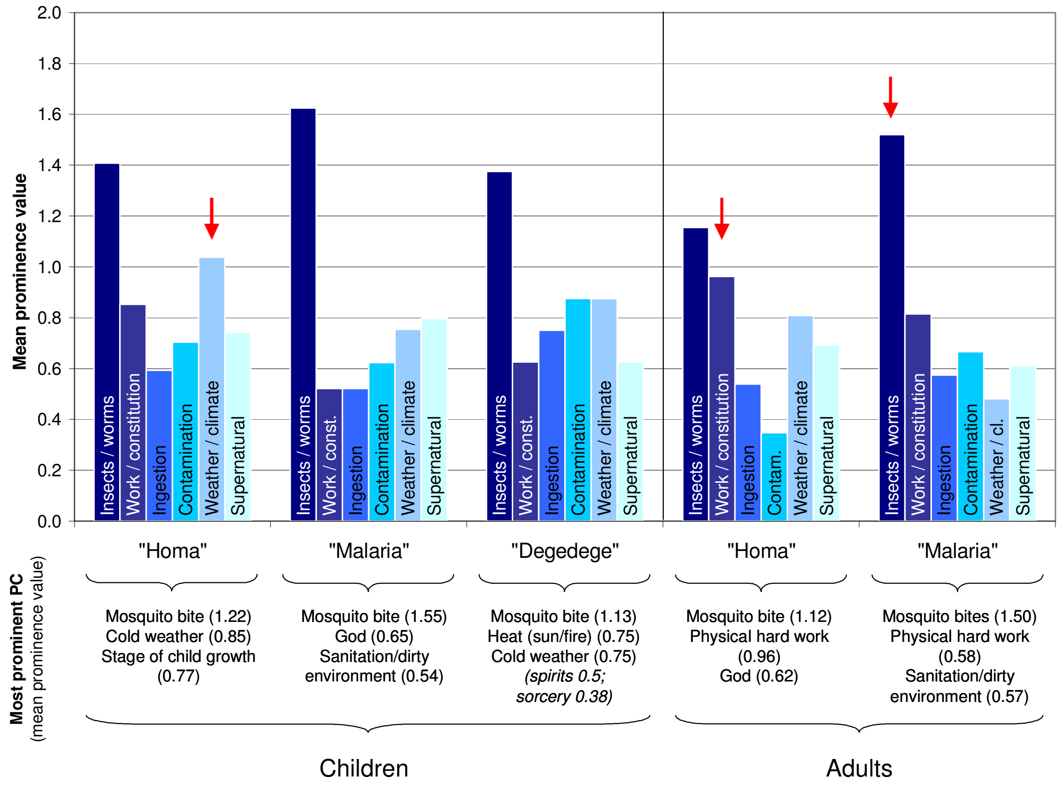

Figure A2: Perceived causes (PC) by illness category. Grouped (bars) and most prominent PC.
